# Advanced Meditation Alters Resting-State Brain Network Connectivity Correlating With Improved Mindfulness

**DOI:** 10.3389/fpsyg.2021.745344

**Published:** 2021-11-18

**Authors:** Ramana V. Vishnubhotla, Rupa Radhakrishnan, Kestas Kveraga, Rachael Deardorff, Chithra Ram, Dhanashri Pawale, Yu-Chien Wu, Janelle Renschler, Balachundhar Subramaniam, Senthilkumar Sadhasivam

**Affiliations:** ^1^Department of Radiology and Imaging Sciences, Indiana University School of Medicine, Indianapolis, IN, United States; ^2^Department of Anesthesia, Critical Care and Pain Medicine, Sadhguru Center for a Conscious Planet, Beth Israel Deaconess Medical Center, Boston, MA, United States; ^3^Department of Radiology, School of Medicine, University of Louisville, Louisville, KY, United States; ^4^Department of Anesthesia, Indiana University School of Medicine, Indianapolis, IN, United States

**Keywords:** meditation, Samyama, Isha yoga, mindfulness, fMRI, brain networks, salience network, default mode network

## Abstract

**Purpose:** The purpose of this study was to investigate the effect of an intensive 8-day Samyama meditation program on the brain functional connectivity using resting-state functional MRI (rs-fMRI).

**Methods:** Thirteen Samyama program participants (meditators) and 4 controls underwent fMRI brain scans before and after the 8-day residential meditation program. Subjects underwent fMRI with a blood oxygen level dependent (BOLD) contrast at rest and during focused breathing. Changes in network connectivity before and after Samyama program were evaluated. In addition, validated psychological metrics were correlated with changes in functional connectivity.

**Results:** Meditators showed significantly increased network connectivity between the salience network (SN) and default mode network (DMN) after the Samyama program (*p* < 0.01). Increased connectivity within the SN correlated with an improvement in self-reported mindfulness scores (*p* < 0.01).

**Conclusion:** Samyama, an intensive silent meditation program, favorably increased the resting-state functional connectivity between the salience and default mode networks. During focused breath watching, meditators had lower intra-network connectivity in specific networks. Furthermore, increased intra-network connectivity correlated with improved self-reported mindfulness after Samyama.

**Clinical Trials Registration:** [https://clinicaltrials.gov], Identifier: [NCT04366544]. Registered on 4/17/2020.

## Introduction

Meditation is increasingly being recognized as an effective method to improve psychological wellbeing. A 2014 meta-analysis of 47 trials found that meditation could lower depression, anxiety, pain, and stress/distress, as well as improve mental health-related quality of life (Goyal et al., 2014). Research has also revealed meditation-related structural and physiological changes in the brain and nervous system (Lazar et al., 2005; Hölzel et al., 2011; Dodich et al., 2019; Yang et al., 2019). Recently, we showed that Samyama program, an intensive meditation program significantly and effectively reduced depression, anxiety while improving physical health (Sadhasivam et al., 2021). This study aimed to demonstrate functional brain changes in Samyama meditators before and after the program, in addition to correlating the functional changes to improved mental health.

Anxiety has been associated with changes in brain activity (Bishop, 2007) such as reduction in prefrontal activity (Bishop, 2009) and alteration in the default mode network (Zhao et al., 2007). Impairment of the default mode network has also been linked to social phobia (Gentili et al., 2009). Positive emotions have also been linked to specific regions. For example, one study showed that relative happiness is correlated with rostral anterior cingulate cortex gray matter density (Matsunaga et al., 2016) and remembering happy events has been linked to activation in the anterior cingulate cortex, prefrontal cortex, and insula (Suardi et al., 2016). Functional connectivity was greater for reward, motivation and emotion regulation network in groups who were “in love” compared to those who were single or ended a relationship (Song et al., 2015). Taken together, this demonstrates the utility of neuroimaging in understanding brain processes involved in both positive and negative emotions. Neuroimaging can therefore give us further insight into brain networks affected by meditation.

The Yoga Sutras, a comprehensive set of ancient texts about yoga written by Patanjali, describe 8 limbs or branches of yoga. These include: (1) Yama (ethical standards), (2) Niyama (self-discipline), (3) Asana (postures), (4) Pranayama (breath control), (5) Pratyahara (withdrawal from senses), (6) Dharana (concentration), (7) Dhyana (contemplation), and (8) Samadhi (union). A combination of the last three is referred to as a process called Samyama. Though Samyama has been around for thousands of years, it has not been scientifically investigated until now. Samyama Program is an 8-day silent residential meditation experience offered by the international non-profit Isha Foundation. Samyama is a rigorous meditation program offered by Isha Foundation for the general population, requiring a substantial number of prerequisite programs and preparation to attend. Preparation to participate in the program requires about 2 months of vegan diet and daily practice of hatha yoga (physical postures), kriya yoga (breathing and sound), and Shoonya meditation (conscious non-doing).

In our recent study, Samyama participants had reduced depression and anxiety and improved subjective well-being scores and health biomarkers (HbA1c, body weight, and lipid profile) compared with their baseline values, and compared to their household non-meditator controls. Participants in this study also mentioned improvements in joy, mindfulness, resilience, and vitality (Sadhasivam et al., 2021). Participants at an Inner Engineering (IE) program, a comprehensive beginner-level yoga program, reported lower levels of perceived stress and higher general well-being after practice (Peterson et al., 2017). Another study demonstrated increased anandamide levels for participants in the Bhava Spandana Program (BSP), a second level meditation retreat (Sadhasivam et al., 2020). Gamma power was shown to increase during three types of meditation (Braboszcz et al., 2017), one of which is Shoonya meditation. Shoonya meditation is a conscious process of non-doing. It is taught in combination with Shakti Chalana Kriya, a breath-based yogic practice (pranayama). Other Isha yoga and meditation programs have also shown benefits such as improved visual plasticity (Braboszcz et al., 2013) and improved cardiac function (Selvaraj et al., 2008; Muralikrishnan et al., 2012). The Shoonya Program, IE and BSP are all prerequisites for the Samyama Program.

Blood oxygen level dependent (BOLD) functional magnetic resonance imaging (fMRI) is a commonly utilized technique to assess brain activity (Bandettini et al., 1992; Ogawa et al., 1992). This technique indirectly measures brain activity by detecting changes in relative blood concentrations of blood oxygen and deoxyhemoglobin (dHb). During task-related neuronal activation at localized regions, increases occur in cerebral blood flow which results in local reduction in deoxyhemoglobin and increase in local oxyhemoglobin. The blood oxygenation level-dependent (BOLD) MRI contrast is dependent on changes in dHb, which acts as an endogenous contrast enhancing agent and serves as the source of the signal for fMRI (Faro et al., 2017). However, there are spontaneous temporally synchronized fluctuations in brain neuronal activity at rest, that are referred to as the resting state networks (Biswal et al., 1997). More than 20 resting state functional networks have been described so far; amongst those, interplay between three networks – the salience, default mode, and frontoparietal (executive) networks, are thought to be important for understanding mechanisms associated with meditation (Raffone et al., 2019).

Various studies on meditative practices like Soham (Guleria et al., 2013), Buddhist tradition of Samatha (Wallace, 2001), Kundalini yoga (Yang et al., 2016), Zen (Ritskes et al., 2003), and Transcendental Meditation (Mahone et al., 2018) practices have shown differences in their brain activation centers (Mishra et al., 2017). Following various meditation techniques, activity is relatively commonly seen in the dorsolateral prefrontal cortex (dl-PFC) (Ritskes et al., 2003), anterior cingulate cortex (ACC) (Tang et al., 2015b; Mahone et al., 2018), and left prefrontal cortex (PFC) (Baerentsen et al., 2010). Brain network connectivity has been shown to be impacted by meditation (Brewer et al., 2011; Hasenkamp and Barsalou, 2012).

With the reported psychological benefits of advanced meditation retreats (Sadhasivam et al., 2020, 2021), it is important to assess the physiological impact of these programs on the brain with advanced meditation program, Samyama. This novel study focused on assessing the changes in functional connectivity before and after the Samyama Program using resting-state fMRI (rs-fMRI) besides correlating the connectivity changes with improved mental wellbeing.

## Materials and Methods

### Samyama Participant Recruitment

These subjects were a subset of the participant group used for an earlier study (Sadhasivam et al., 2021). The Isha Institute of Inner Sciences (McMinnville, TN, United States) provided a registration list for the April 2018 Samyama Program. Each applicant was then individually assessed by an Isha Foundation instructor for suitability to attend the program. The requirement for participation in the Samyama retreat included prior completion of 4 Isha programs (Inner Engineering, Bhava Spandana Program, Shoonya Meditation, and Yogasanas) and a commitment to continue preparatory practices 2 months before the Samyama retreat.

Study eligibility criteria included: Samyama participant and interested cohabitating spouse/partner, at least 18 years of age. Exclusion criteria were: inability to read or comprehend the consent form; subjects with medical conditions in which blood sampling would be contraindicated (e.g., severe anemia); active use of marijuana, opioids, or related drugs; use of antibiotics or probiotic/prebiotic supplements within 60 days of enrollment; participants living outside of the country.

### Study Approvals

The study was reviewed and approved by the Institutional Review Board of the Indiana University School of Medicine. Participants and controls provided electronic informed consent.

### Samyama Participant Dietary Requirements

As part of the Samyama preparatory process (60 days before the program), meditators were required to follow a vegan diet with at least 50% raw foods consumed. They were encouraged to avoid foods which may be considered “negative pranic,” or negative to life energy, including garlic, onion, chili, eggplant, asafoetida, coffee, and tea. Additionally, use of alcohol, cigarettes, stimulants, and illicit drugs was discouraged.

### Samyama Participant Practice Requirements

Samyama participants, also referred as meditators, were asked to perform the following practices daily for the 60-day preparation period. These include kriya yoga practices (Shakti Chalana Kriya and Shambhavi Mahamudra Kriya), hata yoga (Surya Kriya and Yogasanas), Shoonya meditation twice a day, Sukha Kriya and Arda Siddhasana for at least 1 h per day. Kriya yoga practices are combinations of posture, breath, and sound that are meant to purify and enhance the flow of one’s energies while simultaneously increasing general stability. Hata yoga practices consist of postures, meant to improve flexibility and strengthen the body. Shoonya meditation is a process of conscious non-doing. Sukha Kriya consists of alternate nostril breathing which leads to regulation of breath. Ardha Siddhasana is a posture in which one sits cross-legged with the heel of the left foot placed at the perineum.

### Samyama Program

During the program, participants were to remain silent for the entire 8-day duration of the program. The program hall was closed to external influences. No specific instructions or programs were given to the controls, and controls did not practice any meditation.

### MRI

All MR imaging was performed on a Siemens PRISMA 3.0 Tesla Scanner (Siemens, Erlangen, Germany) using a 32-channel head coil. Images acquired included anatomic T1-weighed 3D magnetization-prepared rapid acquisition with gradient echo (MPRAGE; repetition time / echo time [TR/TE] = 2010/2.91 ms, flip angle = 9°, field of view = 192 × 174 mm, 192 sagittal slices, isotropic voxel size of 1 mm) and BOLD rs-fMRI with a gradient-echo planar imaging (EPI) sequence (Axial, TR = 760 ms, TE = 29 ms, flip angle = 54°, 55 slices, field of view [FOV] = 220 × 220 mm^2^, isotropic voxel 2.5 mm, simultaneous multi-slice [SMS] factor 5, 790 volumes). A spin-echo-EPI with reverse phase encoding and matched imaging parameters was also performed for geometric distortion correction.

For both meditator and control groups, the first run of the fMRI was performed at rest. The second run of the fMRI was performed with instructions to focus on their breathing technique, which is a part of the meditation practice.

### Functional Magnetic Resonance Imaging Processing

After visual assessment of quality of the anatomic and BOLD data, fMRI was preprocessed using the standard pipeline with FMRIB Software Library (FSL; Oxford, United Kingdom) (Jenkinson et al., 2012). Fieldmap correction was performed using FSL topup (Andersson et al., 2003; Smith et al., 2004).

After initial preprocessing was done in FSL, the rest of the preprocessing and fMRI analysis was performed with CONN Toolbox (Cambridge, MA, United States) (Whitfield-Gabrieli and Nieto-Castanon, 2012; Nieto-Castanon, 2020). Functional MRI data was realigned using the realign & unwarp function in SPM12 (Andersson et al., 2001). For outlier detection, we used a 97th percentile with a global signal *z*-value threshold of 5 and subject motion threshold of 9 mm. Functional and structural MRI data were then normalized to the standard Montreal Neurological Institute (MNI) T1 template using a direct normalization process. Data was segmented into gray matter, white matter, and cerebrospinal fluid (CSF) (Ashburner and Friston, 1997, 2005). Isotropic resolution of 1 mm for structural images and 2 mm for functional images were used. Next, the data was smoothed using spatial convolution with a Gaussian kernel of 8 mm full width half maximum (FWHM) (Nieto-Castanon, 2020).

Denoising involved removing of noise from white matter and CSF (Behzadi et al., 2007; Chai et al., 2012), estimated subject motion parameters including 3 translation and 3 rotation parameters (Friston et al., 1996), scrubbing (Power et al., 2014), and session effects. For temporal band pass filtering, the lower frequency threshold was 0.008 Hz and the upper frequency threshold was 0.09 Hz. Filtering was performed after regression to avoid mismatch in nuisance regressor procedure (Hallquist et al., 2013; Nieto-Castanon, 2020).

Connectivity was assessed between regions of interest (ROIs). ROI-to-ROI analysis was performed for structures in predetermined networks (see “Brain Networks” section). Comparisons were made within program participants (meditators) before and after the program for both resting-state and focused breathing conditions and corrected for multiple comparisons using FDR threshold of <0.05. Age, gender, and prior participation in the program were entered as co-variates of no interest.

### Brain Networks

Four brain networks were studied – default mode network (DMN), salience network (SN), frontoparietal network (FPN), and dorsal attention network (DAN). The default mode network includes the medial prefrontal cortex (mPFC), posterior cingulate cortex, precuneus, and angular gyrus and is involved with reflective processes (Buckner et al., 2008; Andrews-Hanna, 2012). The salience network primarily includes the anterior cingulate cortex and the anterior insula (AI) and is involved with filtering and prioritizing signals received from external cues (Menon and Uddin, 2010). This network also plays an important role in switching between central executive and default mode networks (Sridharan et al., 2008). The frontoparietal network, also known as the central executive network (CEN), primarily includes the dorsolateral prefrontal cortex (dl-PFC), and posterior parietal cortex (PPC) and is involved with executive functions and cognitive control (Marek and Dosenbach, 2018). Finally, the dorsal attention network includes the intraparietal sulcus (IPS) and front eye fields (FEF) and is involved with voluntary attention (Kincade et al., 2005).

### Psychological Factors

Psychological scores were taken from a subset from a previous study (Sadhasivam et al., 2021). Scores for anxiety (Pilkonis et al., 2011), depression (Andresen et al., 1994), mindfulness (Brown and Ryan, 2003; Osman et al., 2016), joy (Shiota et al., 2006), vitality (Bostic et al., 2000), and resilience (Smith et al., 2008) were accessed with validated surveys. Psychological data was normalized and entered as secondary co-variates in the analysis.

### Statistical Analysis

Statistical analysis was performed with CONN Toolbox (Whitfield-Gabrieli and Nieto-Castanon, 2012). Connectivity between ROIs were assessed using a general linear model. Output values included a t-stat with degrees of freedom, uncorrected *p*-value (p-unc), and a false discovery rate *p*-value (p-FDR) when corrected for multiple comparisons. Greater positive t scores indicated stronger functional connectivity between regions while greater negative scores indicated weaker functional connectivity between regions.

Three sets of comparisons were performed – (A) comparisons within the meditator group before and after the program, (B) comparisons between meditators and controls at each time point, and (C) comparisons within the meditator group with psychological scores before and after the program. Before-after comparisons were performed with the after condition greater than the before. Comparisons between meditators and controls were performed with meditators greater than controls. Comparisons with psychological scores were also compared with the after condition greater than before. A corrected *p*-value of less than 0.05 was considered significant.

## Results

### Demographics

**FIGURE 1:**
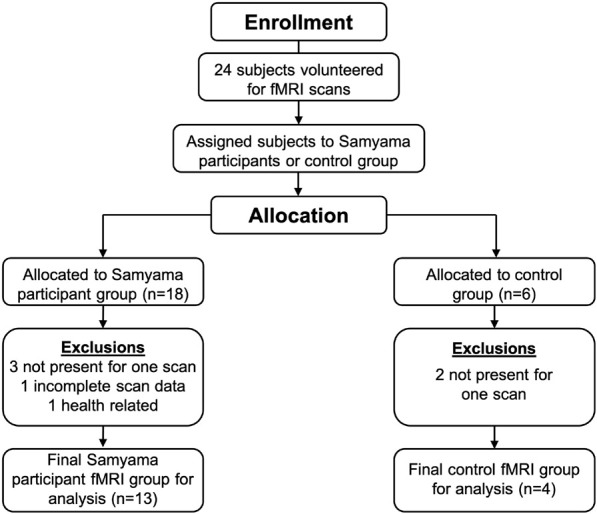
24 participants initially enrolled with 13 meditators and 4 controls included in the final analysis.

We recruited 24 study subjects, 18 Samyama participants (meditators) and 6 controls. In the final analysis, 13 Samyama meditators (8 men and 5 women), and 4 controls (2 men and 2 women) were included (Figure 1). The reasons for exclusions are included in a Consort diagram (Figure 1). Demographic data is shown in Table 1.

**TABLE 1:** Demographic data of Samyama meditators and controls.

	Meditators (*n* = 13)	Controls (*n* = 4)
**Gender**		
Male	8	2
Female	5	2
Age in years (SE)	34.9 (3.6)	62.8 (4.3)
**Prior participation**		
Yes	4	1
No	9	3

### Meditator Functional Magnetic Resonance Imaging Networks Altered After Samyama

**FIGURE 2:**
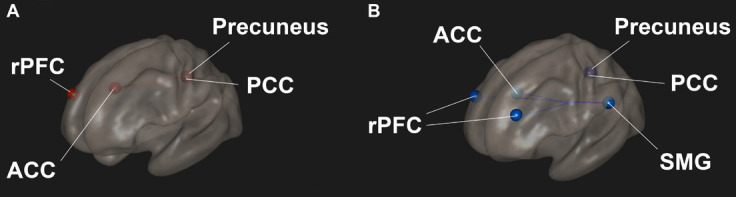
Changes in functional connectivity was observed in meditators and comparisons were made before and after the Samyama program. **(A)** Functional connectivity was increased between the anterior cingulate cortex (ACC) of the salience network and posterior cingulate cortex (PCC) and precuneus of the default mode network (DMN) in the resting state condition. The PCC also had increased connectivity to the rostral prefrontal cortex (rPFC). **(B)** Functional connectivity was decreased within the salience network between the supramarginal gyrus (SMG) and ACC and rPFC in the focused breathing condition. Red indicates increased connectivity and blue indicates decreased connectivity.

Resting state functional connectivity between regions in the SN and DMN were significantly altered in participants after the Samyama program compared to pre-Samyama (Figure 2A). Intra-network connectivity (connectivity between ROIs in the same network) within the SN and DMN was reduced (Figure 2B) during focused breath watching after Samyama program compared to pre-Samyama. In controls, there were no significant changes in functional connectivity for both resting state and focused breathing conditions between the two time points (data not shown). Both resting state and focused breathing data is summarized in Table 2. These results demonstrate connectivity changes with the salience and default mode networks after Samyama and these changes differ based on the resting state and breath watching.

**TABLE 2:** Comparison in meditator functional connectivity from before and after Samyama program.

ROI (Network)	ROI (Network)	T-stat	p-unc	p-FDR
**Meditators resting state**				
ACC (Salience)	PCC (DMN)	4.73	0.0005	0.0047
ACC (Salience)	Precuneus (DMN)	5.09	0.0003	0.0047
rPFC-R (Salience)	PCC (DMN)	5.32	0.0002	0.0017
rPFC-R (Salience)	Precuneus (DMN)	5.98	0.0001	0.0012
**Meditators focused breathing**				
SMG-L (Salience)	rPFC-R (Salience)	–3.8	0.0025	0.0293
SMG-L (Salience)	rPFC-L (Salience)	–3.51	0.0043	0.0293
SMG-L (Salience)	ACC (Salience)	–3.47	0.0046	0.0293
PCC (DMN)	Precuneus (DMN)	–3.9	0.021	0.0404
Precuneus (DMN)	Precuneus (DMN)	–3.9	0.021	0.0404

*Regions of interest (ROI) – anterior cingulate cortex (ACC), posterior cingulate cortex (PCC), rostral prefrontal cortex (rPFC), precuneus, and supramarginal gyrus (SMG). Brain networks – salience network and default mode network (DMN). A p-FDR < 0.05 was considered significant. Positive t-stat indicates increased connectivity while a negative t-stat indicates decreased connectivity.*

**FIGURE 3:**
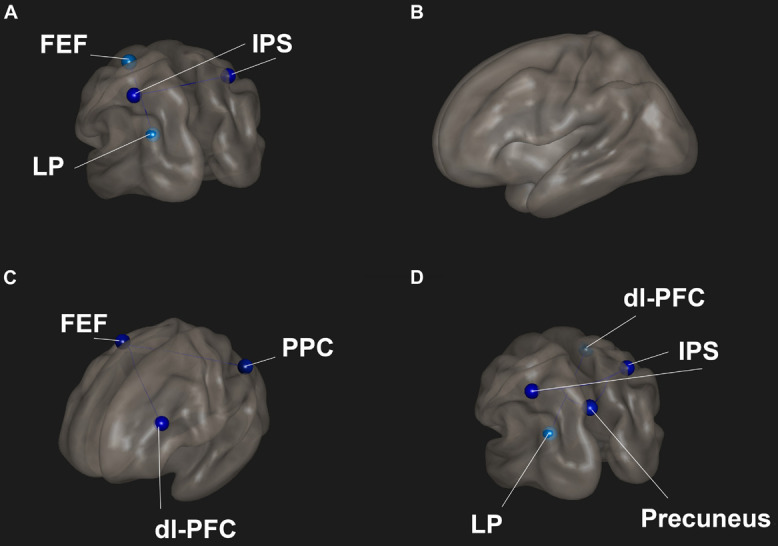
Functional connectivities were compared between meditators and controls for each condition. **(A)** Resting-state pre-program – meditators had decreased connectivity between the front eye fields (FEF) and lateral parietal (LP) lobe and between the left and right intraparietal sulci (IPS). **(B)** Focused breathing pre-program – no significant difference between the groups. **(C)** Resting-state post program – meditators had significantly reduced connectivity between the FEF and dorsolateral prefrontal cortex (dl-PFC) and posterior parietal cortex (PPC). **(D)** Focused breathing post program – meditators had significantly reduced connectivity between the dl-PFC and LP and the IPS and posterior cingulate cortex (PCC). Red indicates increased connectivity and blue indicates decreased connectivity.

Meditator scans showed lower functional connectivity between the DAN and DMN and within the DAN compared to controls (Figure 3A). There were no significant differences between meditators and controls in the focused breath watching condition before Samyama program (Figure 3B). Following completion of the Samyama, resting state scans showed less connectivity between the DAN and FPN (Figure 3C). Focused breath watching scans showed less connectivity between the DMN and DAN and FPN. Additionally, there was reduced intra-network connectivity in the DAN (Figure 3D). This data is summarized in Table 3. This data demonstrates that meditators had less functional connectivity compared to controls between the dorsal attention and default mode networks and dorsal attention, default mode, and frontoparietal networks.

**TABLE 3:** Comparison between meditators and controls for functional connectivity at both time points and conditions.

ROI (Network)	ROI (Network)	T-stat	p-unc	p-FDR
**Pre program resting state**				
FEF-L (DAN)	LP-L (DMN)	−3.62	0.0025	0.0476
IPS-R (DAN)	IPS-L (DAN)	−3.65	0.0024	0.0447
**Post program resting state**				
FEF-R (DAN)	dl-PFC-L (FPN)	−4.64	0.0003	0.0033
FEF-R (DAN)	PPC-L (FPN)	−4.6	0.0003	0.0033
**Post program focused breathing**				
Precuneus (DMN)	IPS-R (DAN)	−3.62	0.0025	0.0405
Precuneus (DMN)	IPS-L (DAN)	−3.36	0.0043	0.0405
LP-L (DMN)	dl-PFC-R (FPN)	−3.86	0.0015	0.0294
IPS-R (DAN)	Precuneus (DMN)	−3.62	0.0025	0.0258
IPS-R (DAN)	PCC (DMN)	−3.58	0.0027	0.0258
IPS-R (DAN)	IPS-L (DAN)	−3.09	0.0074	0.0469

*Regions of interest (ROI) – front eye fields (FEF), lateral parietal (LP), intraparietal sulcus (IPS), dorsolateral prefrontal cortex (dl-PFC), posterior parietal cortex (PPC), and precuneus. Brain networks – dorsal attention network, default mode network (DMN), and frontoparietal network (FPN). A p-FDR < 0.05 was considered significant. Positive t-stat indicates increased connectivity while a negative t-stat indicates decreased connectivity.*

### Mindfulness Score Correlates With Changes in Functional Connectivity

**FIGURE 4:**
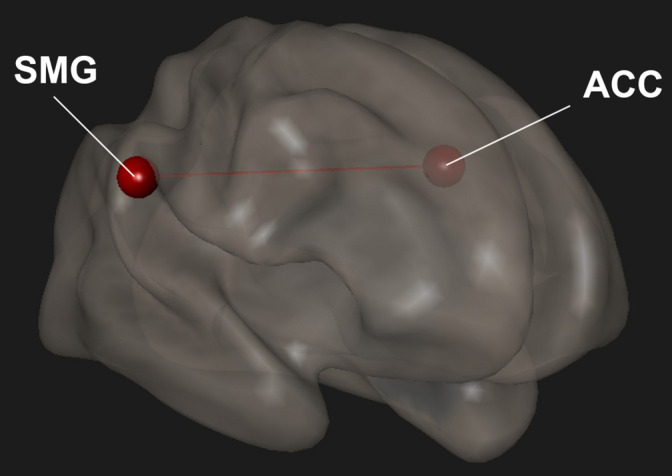
In meditators completing surveys, changes in mindfulness scores were correlated with changes in functional connectivity. There was increased functional connectivity between the anterior cingulate cortex (ACC) and supramarginal gyrus (SMG). Red indicates increased connectivity and blue indicates decreased connectivity.

We previously showed that Samyama participants had reduced anxiety and depression and increased mindfulness, joy, vitality, and resiliency (Sadhasivam et al., 2021) compared to their pre-Samyama baseline values. Improved mindfulness scores correlated with increased functional connectivity within the SN between the SMG and ACC (*p* < 0.05) (Figure 4). We did not observe any significant correlation between the fMRI changes and scores for anxiety, depression, joy, vitality, and resilience (Table 4).

**TABLE 4:** Correlation between changes in mindfulness scores and functional connectivity in meditators.

ROI (Network)	ROI (Network)	T-stat	p-unc	p-FDR
SMG-R (Salience)	ACC (Salience)	3.88	0.0019	0.0359

*Regions of interest (ROI) – supramarginal gyrus (SMG) and anterior cingulate cortex (ACC). Brain network – salience network. A p-FDR < 0.05 was considered significant. Positive t-stat indicates increased connectivity while a negative t-stat indicates decreased connectivity.*

## Discussion

This novel study demonstrates that Samyama, an intensive silent meditation program, increased the resting-state functional connectivity between the salience and default mode networks. Furthermore, increased intra-network connectivity correlated with improved self-reported mindfulness after Samyama. The Samyama meditators showed significant changes in functional connectivity and while no changes were observed within the non-meditator control group. Interestingly, the changes within the meditator group differed based on task condition at resting state and focused breath watching. During the focused breath watching after the Samyama program, the meditators had less functional connectivity than controls between the DAN, DMN, and FPN (also referred to as CEN) for 3 of the 4 points tested, demonstrating specific and dynamic meditation-related changes in the brain.

In meditators, during the resting state, we found significant increases in functional connectivity between regions in the salience network and default mode networks, specifically between the ACC and PCC and precuneus. We were also able to correlate improved mindfulness scores to increased connectivity with the SN. This result corresponds with previous findings in literature where increased mindfulness from meditation was linked with increased functional connectivity between the SN and DMN (Doll et al., 2015).

During focused breath watching, functional connectivity within the salience and default mode networks uniquely reduced after Samyama compared to the baseline and resting state, demonstrating the ability of the meditators to voluntarily and dynamically influence and control certain brain connectivity based on meditation-related specific tasks such as focused breath watching. This can potentially explain improved mindfulness, concentration, cognitive control and executive function at the resting state and ability of the meditators to focus on specific meditation-related tasks and reduce connectivity in certain brain networks compared to the resting state. The focused breathing condition is a more internalized state than the resting state. Internalized states, such as having one’s eyes closed, have shown to elicit different responses in brain functional connectivity than the eyes-open state (Agcaoglu et al., 2020; Weng et al., 2020). Furthermore, there was greater connectivity within the DMN and lower connectivity within the SN in the eyes-closed group (Costumero et al., 2020). Considering that the focused-breathing condition is a more internalized state, it is interesting that our results showed less connectivity within the DMN after Samyama. Since the task was *focused* breathing, this could explain why connectivity within the DMN was reduced; the DMN has been previously linked to states of mind-wandering (Mason et al., 2007; Christoff et al., 2009; Poerio et al., 2017).

This study has shown that the ACC had increased connectivity in meditators and linked to improved mindfulness scores. The ACC is a structure that has been linked to increased connectivity due to improvements in attention in prior meditation studies (Tang et al., 2015a). Meditative practices were also linked to increased blood flow to this region (Zeidan et al., 2014; Tang et al., 2015b; Mahone et al., 2018). While the role of the ACC has been debated, it is generally thought to be involved in cognitive control (Ridderinkhof et al., 2004), happiness (Matsunaga et al., 2016; Suardi et al., 2016), attention (Kim et al., 2016; Wu et al., 2017), and empathy (Lockwood et al., 2015). It is associated with the salience network SN but has also been linked with executive functions (Carter et al., 1999).

Another region linked with executive functions is the prefrontal cortex which is associated with concentration, decision-making and awareness (Allman et al., 1993) and is a part of the CEN (Menon, 2011). Meditation has shown to suppress DMN and increase functional connectivity between DMN and CEN (Bauer et al., 2019) and CEN and attention networks (Taren et al., 2017). Bauer et al. (2019) suggested a neural mechanism by which the CEN negatively regulates the DMN by showing gradual reconfiguration in DMN and CEN in meditation state and post meditation state (state to trait) by means of positive diametric activity (PDA); the reported psychological well-being in long-term meditators was likely due to trait changes caused by reconfiguration and recalibration of network structure, or homeostatic plasticity (Davis, 2013; Hellyer et al., 2017). This in turn causes reductions in DMN activity and stronger anti-correlated coupling between CEN and DMN (Mooneyham et al., 2017; Marusak et al., 2018).

Contrary to the early dichotomized view of the DMN and CEN regions representing dorsal-caudal “cognitive” and ventral-rostral “affective” subdivisions, both regions have been shown to make key contributions to emotional processing (Etkin et al., 2011). Positive emotions, which regulate and diminish negative emotions, have been associated with activation in the sub genual ACC, ventromedial prefrontal cortex (PFC) and pre-genual ACC (Wager, 2008). The medial prefrontal (mPFC) cortex and ACC are activated with not only negative emotions, but also positive emotions. Empathy for others experiencing pain and one’s experience of pain activate the dorsal ACC/mPFC (Lamm et al., 2011). Lesions of the dorsal ACC serve in treating chronic pain (Wilkinson et al., 1999). Endogenously driven analgesia, by means of the “placebo effect,” has been closely tied to the pre-genual ACC, which is presumed to modulate regions that generate opioid-mediated anti-nociceptive responses, such as the amygdala and periaqueductual gray (Petrovic et al., 2002; Eippert et al., 2009).

Interactions between the DMN, SN and the CEN are thought to be key to understanding the mechanism of action of meditative practices on the brain (Raffone et al., 2019). In this study, we demonstrate unique and different changes in functional connectivity in the resting state and focused breath watching. The latter produces a more meditation-related task-based changes in functional brain connectivity compared to the resting state; therefore, specific tasks need to be included when analyzing resting and meditation-related task-related changes in fMRI. Even opening and closing eyes has shown to impact salience and default mode networks (Costumero et al., 2020). Additionally, experienced meditators would have different regions of brain activation compared to novices (Baron Short et al., 2010) and long-term meditators have shown significant neural changes (Holzel et al., 2007; Luders et al., 2011; Fayed et al., 2013; Engen et al., 2018). We have uniquely demonstrated increased and decreased intra-network connectivity in advanced meditators during the resting state and meditation-related focused breath watching. Importantly, improved mindfulness scores correlated with the functional brain connectivity changes after the Samyama program.

We previously showed that participation in the Samyama program decreased negative psychological states and boosted positive psychological states (Sadhasivam et al., 2021). Here, we were able to observe significant changes in functional connectivity at rest and these changes could help explain the positive findings in the previous study. This is significant because it suggests that the effects of the Samyama program seem to be maintained outside of a meditative practice and provides a physiological measurement. It is also important to note that these changes occurred over a relatively short period of 8 days.

The strengths of this study are that it objectively demonstrates significant changes after the Samyama program in the meditator group. Criteria for determining significance was stringent as it accounted for multiple comparisons. It was also able to show, with significance, different changes based on task condition. Finally, it was able to correlate changes in mindfulness scores to changes in functional connectivity. Taken together, this study helps advance our understanding of the impact of meditation on brain networks.

This study did have some limitations. There were a relatively small number of meditators in this study due to a limited number of Samyama participants throughout the USA that were willing to visit Indiana University MRI scanner twice, before and after the Samyama program. Though the number of meditators in the final analysis is relatively small, we did pre- and post-Samyama MRIs in the meditators, having them as their own controls. Second, Samyama meditators were involved with 2 months of intensive preparation before experiencing the 8-day program. Therefore, the meditator group may already have some changes prior to the program which may not be reflected in the control group. We report statistically significant, objective and consistent changes in the meditators post-Samyama. The task related changes (during focused breath watching) were consistently and objectively different after Samyama compared to pre-Samyama values. Moreover, to avoid false positive findings, we used a conservative statistical approach with stringent corrections for multiple comparisons, when we observed significant differences. It was a functional study; therefore, to avoid misregistration, clear instructions were given regarding the procedure and expected patient experiences (e.g., MRI machine noise, commands to follow and for what duration) and questions were answered. The commands were given in the same way during the image acquisition to reproduce highly specific and sensitive information. Despite that, individual variations cannot be eliminated regarding patient meditation inside the scanner versus outside. Ear plugs were given during the fMRI scanning to blot out the machine noise. However, its effect on the qualitative measure of individual meditation could not be eliminated (Travis et al., 2020). The images had minimal noise, thus favoring an adequate imaging study, based on which these inferences have been made.

Another limitation was the number of consenting controls. Because of a small number of controls, they were age matched. The average age of meditators was in the mid 30 s while the average age of controls was in the 60 s. This is a significant consideration since functional connectivity has shown to change with age (Betzel et al., 2014; Geerligs et al., 2015). Therefore, findings from comparisons made between the meditator and control groups should be approached with some caution. To minimize potential differences due to other factors such as age, we used controls and meditators their own controls as we did fMRI scans before and after the Samyama program. It is important to note that the MRI scans were obtained before and after Samyama program 3 years ago. At this point, we are unable to re-create the conditions to obtain suitable controls in terms of timing of scans similar to meditators and artifacts associated with harmonization of fMRI even if we were to use the same MRI machines and protocols. Despite these limitations, this study provides novel insight into brain mechanisms before and after Samyama program during the resting state and focused breath watching and demonstrates correlations with improved mindfulness after Samyama. Future studies using a larger sample size and proper age-matched controls can further investigate functional connectivity changes in different regions during resting states and meditative practices, in addition to correlating with other psychological improvements associated with advanced meditation.

## Conclusion

Samyama, an 8-day intensive meditation program, favorably influenced the functional connectivity between the salience and default mode networks on meditators compared to their baseline and non-meditator controls. Furthermore, specific brain functional connectivity changes were different at resting state and meditation-related focused breath watching in meditators. This study was also able to correlate changes in functional connectivity to improved mindfulness scores in meditators. Results are consistent with existing literature regarding the observed changes in functional connectivity of the anterior cingulate cortex from meditative processes. Studies with larger sample sizes can further investigate functional connectivity changes in different regions during resting states and meditative practices, in addition to correlating with longer-term and other psychological improvements associated with advanced meditative practices.

## Data Availability Statement

The datasets presented in this article are not readily available because this is a subset of a larger study group and further analysis is currently planned. Raw data will be shared after the analysis is complete. Requests to access the datasets should be directed to SS at ssenthil@pitt.edu.

## Ethics Statement

The studies involving human participants were reviewed and approved by this study was reviewed and approved by the Indiana University School of Medicine Institutional Review Board. All subjects provided written or electronic consent to participate. The patients/participants provided their written informed consent to participate in this study.

## Author Contributions

RV and RR contributed to the study design, data analysis and interpretation, and manuscript drafting and editing. KK and RD contributed to the data analysis. CR and BS contributed to the study design and manuscript preparation. JR contributed to the manuscript drafting and editing. DP contributed to the research coordination, IRB approval and communications, study conduct, and data collection. SS contributed to the study design, conduct, data collection, coordination, manuscript preparation, and arranging funding for this study. All authors have approved the submitted version and have agreed both to be personally accountable for the author’s own contributions and to ensure that questions related to the accuracy and integrity of any part of the work, even ones in which the author was not personally involved, are appropriately investigated, resolved, and the resolution documented in the literature.

## Conflict of Interest

The authors declare that the research was conducted in the absence of any commercial or financial relationships that could be construed as a potential conflict of interest.

## Publisher’s Note

All claims expressed in this article are solely those of the authors and do not necessarily represent those of their affiliated organizations, or those of the publisher, the editors and the reviewers. Any product that may be evaluated in this article, or claim that may be made by its manufacturer, is not guaranteed or endorsed by the publisher.
